# Effects of Climate and Land Use on Different Facets of Mammal Diversity in Giant Panda Range

**DOI:** 10.3390/ani15050630

**Published:** 2025-02-21

**Authors:** Qibing Che, Chunxiao Li, Xuzhe Zhao, Jindong Zhang, Junfeng Tang, Caiquan Zhou

**Affiliations:** 1 Key Laboratory of Southwest China Wildlife Resources Conservation, Ministry of Education, Institute of Ecology, China West Normal University, Nanchong 637009, China; qibing_che@126.com (Q.C.); chunxiaoli2016@163.com (C.L.); xuzhe_zhao@126.com (X.Z.); zhangjd224@163.com (J.Z.); 2 Hubei Key Laboratory of Biologic Resources Protection and Utilization, Hubei Minzu University, Enshi 445000, China

**Keywords:** giant panda, climate, land use, diversity facets, conservation, topographic heterogeneity

## Abstract

Understanding the patterns and drivers of different facets of biodiversity is crucial for biodiversity conservation under global environmental change. In this study, we present the first assessment of the patterns of taxonomic, functional and phylogenetic diversity for 171 mammals in the giant panda range and their associations with climate, land use factors and topographic heterogeneity. We found that functional diversity showed a very different pattern with species richness and phylogenetic diversity. Additionally, mammal assemblages were more functionally diverse but phylogenetically similar than expected by chance in very few regions after controlling for species richness. Furthermore, species richness was positively correlated with topographic heterogeneity, the proportion of forest, mean annual temperature and temperature anomaly and negatively correlated with annual precipitation and precipitation anomaly between current and historical periods, while both functional and phylogenetic diversity are predominantly correlated with climate factors. Specifically, higher functional and phylogenetic diversity was mainly found in regions with a lower proportion of cropland, annual precipitation, mean annual temperature and precipitation anomaly between current and historical periods. These results indicate the large mismatches of driving factors between taxonomic diversity and the other diversity facets and the importance of contemporary climate and land use conditions and climate anomaly between current and historical periods in determining mammal functional and phylogenetic diversity in the giant panda range. Overall, our findings highlight the importance of integrating multiple dimensions of diversity to infer the underlying processes determining the spatial pattern of biodiversity and to better inform conservation management and planning.

## Simple Summary

A crucial part of biodiversity conservation is understanding biodiversity patterns and drivers. By assessing the patterns and determinants of taxonomic, functional and phylogenetic diversity of mammals in the giant panda range, we found that the patterns of functional diversity were inconsistent with species richness and phylogenetic diversity. After controlling for species richness, in very few regions, the functional diversity of mammal assemblages was more diverse than expected by chance, but phylogenetic diversity was more similar. Moreover, contemporary climate and land use conditions and climate anomalies between current and historical periods are the main drivers of mammal functional and phylogenetic diversity in the giant panda range. These results indicate the large mismatches of patterns and driving factors of species richness, functional diversity and phylogenetic diversity, highlighting the importance of integrating multiple dimensions of diversity when informing conservation management and planning.

## 1. Introduction

Biodiversity is causally linked to ecosystem function and services, which are critical to human health and well-being [1]. In recent years, with the increasing concern about the negative impacts of anthropogenic global change on ecosystems [2,3], biodiversity metrics have become important issues [4]. This is largely because the concept of biodiversity itself means the biological variabilities among living organisms in many aspects [5], and therefore, biodiversity metrics can have important implications for biodiversity conservation [6]. Until now, many diversity metrics have been proposed to quantify the biological variabilities among organisms in different respects. The three most commonly used metrics are taxonomic diversity (e.g., species richness), which measures taxonomic differences among species; functional diversity (FD), which measures trait variability among organisms [7]; and phylogenetic diversity (PD), which measures the evolutionary differences or relationships among species [8]. Among the above three facets of biodiversity, species richness has widely been used and has sometimes been used as a surrogate for FD and PD, assuming that species richness can adequately capture both FD and PD [9]. However, there is a growing consensus that both FD and PD can better describe community structure and function compared to species richness [10], and consequently, only considering species richness and neglecting FD and PD may hinder our full understanding of the processes that underlie the spatial and temporal dynamics of biodiversity [11,12,13]. Therefore, FD and PD must be included alongside species richness when studying patterns and determinants of biodiversity and/or developing biodiversity management and conservation strategies [14].

Actually, a number of studies have shown that FD and PD can also provide insights into ecological and evolutionary mechanisms underlying biodiversity patterns. In particular, the geographical distribution of traits and clades can help to reveal what makes some regions hotspots of biodiversity while others exhibit little biodiversity [12,13]. Climate factors, including contemporary and historical climate factors, have been shown to have a remarkably strong association with FD and PD [15,16]. For example, more benign contemporary climate conditions (e.g., suitable temperature and precipitation) can promote richer assemblages, allowing higher levels of diversification of traits and clades [17]. At the same time, long-term climatic stability could also promote a long-term persistence of high diversity due to lower extinction risks [18,19]. In addition to climate factors, land use factors are also important drivers in shaping the spatial patterns of FD and PD [20], mainly by extirpating species with certain traits and/or clades from local assemblages, thus resulting in the loss of FD and PD [21,22]. Furthermore, other environmental factors, such as topographic heterogeneity, are also thought to be related to FD and PD [16,23]. Moreover, a large body of evidence exists for many of these factors’ influence on different facets of biodiversity. However, previous studies focus on these factors separately (e.g., [24]), and systematic evaluations of the effects of these factors on different facets of biodiversity are rare.

Understanding the patterns and drivers of multiple facets of biodiversity is particularly crucial in biodiversity hotspots [13], as many species in these regions are either functional or phylogenetically unique, and deciphering this uniqueness is essential for uncovering large-scale ecological and evolutionary patterns and for protecting core regions and core elements of biodiversity [15,16]. The giant panda (*Ailuropoda melanoleuca*), widely known as an iconic species, is currently distributed in a typical biodiversity hotspot—China’s Sichuan province [25]. This region harbors approximately one-half of the mammals that are distributed in China [26]. Owing to anthropogenic changes in land use patterns (e.g., deforestation or agriculture expansion), many mammals in this region are currently experiencing high extinction risks [27]. Many species, such as large carnivores, have become extinct or functional extinct [28]. Moreover, many studies suggested that these current risks may be exacerbated under future climate change, which would further reduce mammal diversity in this region [29]. Therefore, it is critical to assess the patterns and determinants of multiple dimensions of mammal diversity in this region. However, previous studies mainly focus on species richness [30,31]; a systematic assessment of the influence of climate and land use on different facets of mammal diversity in the giant panda range is rare, limiting our understanding of the underlying factors that drive the distribution patterns of mammal diversity and our ability to develop more effective conservation strategies.

In this study, we aim to explore the impacts of climate, land use factors and topographic heterogeneity on different facets of mammal diversity in the giant panda range. Our specific objectives are as follows: (1) uncover the spatial patterns of taxonomic, functional and phylogenetic diversity of mammals; (2) explore the influences of climate (contemporary temperature and precipitation and climate anomaly since the Last Glacial Maximum climate), land use (proportion of cropland, forest and urban green space) and topographic heterogeneity (standard deviation of elevation) on taxonomic, functional and phylogenetic diversity of mammals in giant panda range. To our knowledge, this is one of the first studies to evaluate the impact of climate and land use on different facets of mammal diversity in the giant panda range, which can inform more effective conservation planning and strategies for protecting giant panda and other mammals in its distribution range.

## 2. Materials and Methods

### 2.1. Study Area and Species Distribution Data

This study was conducted in the Liangshan, Xiaoxiangling, Daxiangling, Qionglai, Minshan and Qingling mountains of western China. This region offers an especially rich system for testing the hypotheses related to the diversity distribution patterns of mammals for several reasons: (i) this region is home to the giant pandas; (ii) as one of the global biodiversity hotspots for conservation, this region also harbors many mammals that are phylogenetically diverse and vary greatly in biological traits [26]; (iii) the distribution of giant panda and other mammals in this region are closely related to climatic-, topographic- and human-related factors [30,32].

The published, expert-drawn distribution maps for 218 mammals in the study area were obtained from the IUCN (International Union for Conservation of Nature; 2017). We used this type of extent-of-occurrence distribution maps rather than the widely used point occurrence records of species, for the latter are often strongly biased taxonomically [33]. To map different facets of the diversity of these mammals, we divided the whole study area into 1711 grid cells of 5 × 5 km^2^. For each spatial scale, each distribution map was overlaid onto each grid cell across the whole region. For each species, if its distribution polygon of a species was intersected with a grid cell, then it was considered to be present in that grid cell, or it was considered to be absent in that grid cell. Since the extent-of-occurrence distribution maps may potentially overestimate the distribution of each species, we further refined the distribution of each species based on the elevation limit and habitat type of each species, both of which are available from the IUCN [27]. Specifically, SRTM data at 250 m resolution obtained from the International Centre for Tropical Agriculture (http://srtm.csi.cgiar.org, accessed on 9 June 2023) and a vegetation map of China (1:1,000,000) obtained from the Resource and Environmental Science Data Platform of China (https://www.resdc.cn, accessed on 9 June 2023) were overlaid onto all the gird cells across the whole study area, respectively. Then, for each species, those gird cells that did not contain their habitat types or were out of their elevation limit were considered as the non-distribution area.

### 2.2. Calculation of Diversity Indices

For each spatial scale, three indices were used to quantify different facets of mammal diversity in each grid cell: species richness and functional and phylogenetic diversity. For each grid cell with two or more species, we calculated functional richness as a metric of functional diversity using the ‘dbFD’ function in the R package ‘FD’ (https://CRAN.R-project.org/package=FD, accessed on 9 June 2023), based on the functional trait data obtained from a recently published traits dataset for Chinese mammals [34]. As an incomplete dataset may lead to uncertain estimates for function diversity [35], 47 mammals with incomplete functional trait data were excluded in the subsequent analysis, while the remaining 171 mammals were retained in calculating species richness, functional diversity and phylogenetic diversity in each grid cell. Specifically, nine traits that are common to all mammals, including hindfoot length (mm), tail length (mm), body length (mm), body mass (g), litters per year, litter size, maturity, generation length and gestation length, were used to calculate functional richness. Similarly, for each grid cell with two or more species, Faith’s phylogenetic diversity [8] was calculated as a measure of phylogenetic diversity using the ‘pd’ function in the R package ‘picante’ (https://CRAN.R-project.org/package=picante, accessed on 9 June 2023), based on the phylogenies obtained from the ‘U.PhyloMaker’ R package [36], which exploits the mega-phylogeny for 5911 mammals worldwide [37].

As the FD/PD are often closely correlated with SR [38], we used null model simulations to produce FD and PD independent from SR. Specifically, following Tordoni [13], we created 999 null communities for each grid cell using the ‘Curveball algorithm’, which can quickly recompile the presence/absence matrix using the randomized species list without changing the total number of species and sites [39]. For each grid cell, the standardized effect size (SES) was then calculated as follows:

$$SES={Metric}_{obs}-{mean}_{null}/{SD}_{null},$$

where ${Metric}_{obs}$ is the observed value of FD or PD in the given grid cell, ${mean}_{null}$ is the mean of the FD or PD values calculated from 999 randomized communities, and ${SD}_{null}$ is the standard deviation of all 999 null values. The positive and negative values of SES indicate that the observed value is higher and lower than the mean expected value of the null values, respectively. Moreover, the SES values greater than 1.96 or less than −1.96 are considered to be over-dispersed and under-dispersed, respectively. Given that we aim to explore the impacts of different drivers in shaping the functional and phylogenetic diversity of mammals across all the grid cells, we used the standardized effect sizes for functional and phylogenetic diversity as response variables for further analyses.

### 2.3. Environmental Predictors of Mammal Diversity

To assess the potential effect of climate and land use on different facets of mammal diversity, we compiled three groups of environmental variables, including current climate, climate anomaly and topographic heterogeneity. For all analyses, we obtained all the variables in their finest available spatial resolution and calculated their mean values to match our grid’s resolution. Current climate was represented by mean annual temperature (MAT) and annual precipitation (AP) during 1981–2010, obtained from the CHELSA (climatologies at high resolution for the earth’s land surface areas) dataset [40]. Climate anomaly was represented by the Last Glacial Maximum anomaly for MAT (LGM MAT anomaly) and AP (LGM AP anomaly) following Guo et al. [16]; that is, the absolute difference of temperature and precipitation between the current and the Last Glacial Maximum climate. The temperature and precipitation data (i.e., mean annual temperature and annual precipitation) for the current and the Last Glacial Maximum were also obtained from the CHELSA dataset. For each grid cell, climate anomaly measures were calculated for all three general circulation models (CCSM4, MIROC-ESM, MPI-ESM-P). To reduce the uncertainty of these climate models, the averaged climate stability values for each grid cell were used for further analyses. Land use was represented by the proportion of ten land use types in each grid cell, including bare land, cropland, forest, grassland, impervious, shrubland, snow/ice, urban green spaces (UGS), water and wetland [41]. Topographic heterogeneity was represented by mean elevation range and standard deviation (SD) of elevation. The elevation data were obtained from the International Centre for Tropical Agriculture (http://srtm.csi.cgiar.org, accessed on 9 June 2023).

To avoid multi-collinearity among the above 16 environmental predictors, we retained only the variables with Pearson’s correlation < |0.7| and variance inflation factors < 5 [42]. Finally, eight variables were selected for further analysis: mean annual temperature, annual precipitation, LGM MAT anomaly, LGM AP anomaly, the proportion of cropland, proportion of forest, proportion of UGS and SD of elevation.

### 2.4. Statistical Analysis

We used Kruskal–Wallis tests to assess whether the three facets of mammal diversity (i.e., taxonomic, functional and phylogenetic diversity) differed among mountains. We further tested the pairwise difference in the above three facets of mammal diversity between mountains by performing Dunn’s tests. Generalized least squares (GLS) regression was used to examine the relationship between the selected predictor variables and different dimensions of mammal diversity (i.e., SR, SES FD and SES PD). We used the GLS model because of its capacity to model data sets that exhibit non-normality and/or auto-correlation [12]. To account for spatial autocorrelation, all GLS models were fitted with an exponential correlation structure using the geographical coordinates of the centroid of each grid cell. Moreover, to assess the relative importance of the selected predictor variables in determining the variation in different dimensions of mammal diversity, all selected predictor variables were centered to zero with a standard deviation of one to derive comparable estimates, using the ‘scale’ function in the R package ‘base’ (Version 3.6.2). All GLS models were run using the ‘gls’ function in the R package ‘nlme’ (Version 3.1-165).

## 3. Results

### 3.1. Geographic Patterns of Taxonomic, Functional and Phylogenetic Diversity

**Figure 1:**
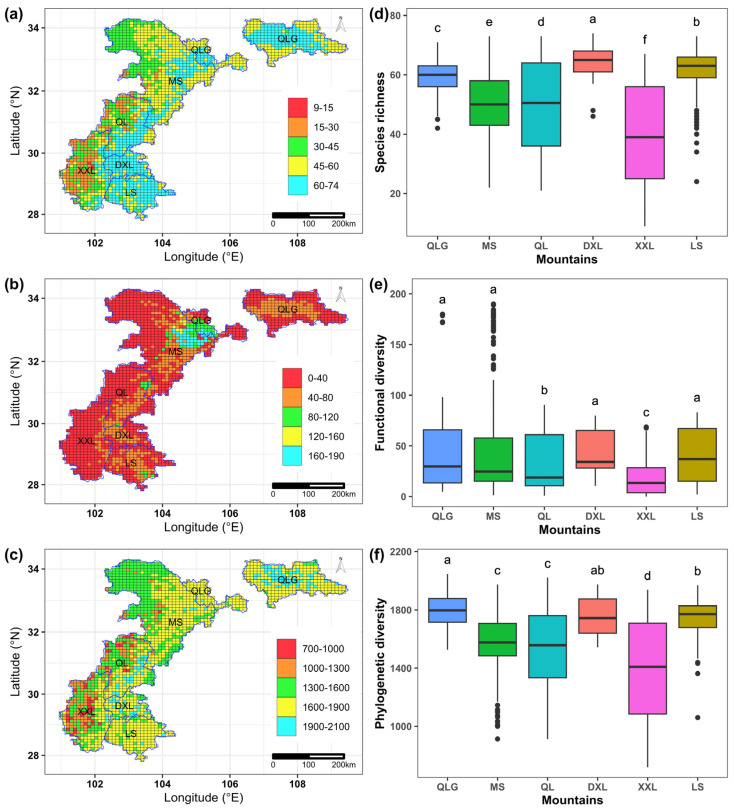
Spatial patterns of (**a**) species richness, (**b**) functional diversity, (**c**) phylogenetic diversity in the whole study area and comparisons of species richness (**d**), functional diversity (**e**), phylogenetic diversity (**f**) among the six mountains in the study area: Qinling (QLG), Minshan (MS), Qionglai (QL), Daxiangling (DXL), Xiaoxiangling (XXL) and Liangshan (LS) mountains. Significance tests were performed using Kruskal–Wallis tests, and Dunn’s tests were used for multiple comparisons. Different superscript letters (a–f) indicate significant differences between mountains.

**Figure 2:**
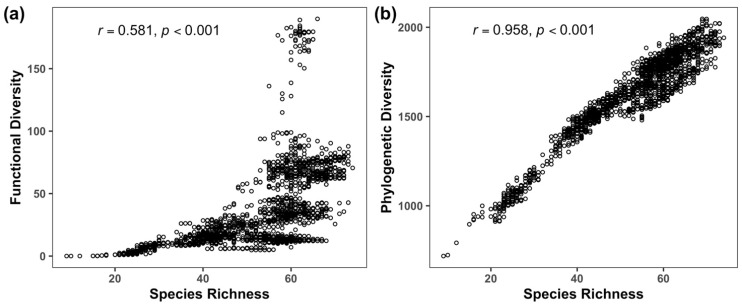
The spatial correlation (**a**) between species richness and functional diversity and (**b**) between species richness and phylogenetic diversity. The spatial correlation tests were performed using a modified *t*-test, which can account for spatial autocorrelation between two non-independent spatial variables.

The mean mammal species richness and functional and phylogenetic diversity across all grid cells in our study area were 51.86 ± 13.19, 35.99 ± 34.03, 1609.83 ± 252.25, respectively. All these three diversity facets decreased from the eastern to the western (Figure 1a–c). Moreover, these three diversity facets showed a very similar spatial pattern with respect to different mountains (Figure 1d–f). Specifically, the top three mountains with the highest species richness and functional and phylogenetic diversity were Qingling (SR = 59.40 ± 5.30; FD = 39.13 ± 31.93; PD = 1798.15 ± 112.14), Liangshan (SR = 61.05 ± 7.83; FD = 41.83 ± 25.13; PD = 1737.79 ± 129.90) and Daxiangling (SR = 64.31 ± 4.89; FD = 41.85 ± 23.16; PD = 1758.22 ± 128.58) mountains, while Xiaoxiangling mountains has the lowest species richness (SR = 39.49 ± 15.08) and functional (FD = 18.09 ± 17.26) and phylogenetic diversity (PD = 1390.25 ± 317.41) (Figure 1d–f). Despite that, the spatial correlation between functional diversity and species richness (*r* = 0.581, *p* < 0.001) is lower than that between phylogenetic diversity and richness (*r* = 0.958, *p* < 0.001), as functional diversity was lower in most areas with high species richness while phylogenetic diversity closely mirrored species richness in the whole study area (Figure 2).

### 3.2. Geographic Patterns of Richness-Controlled Functional and Phylogenetic Diversity

**Figure 3:**
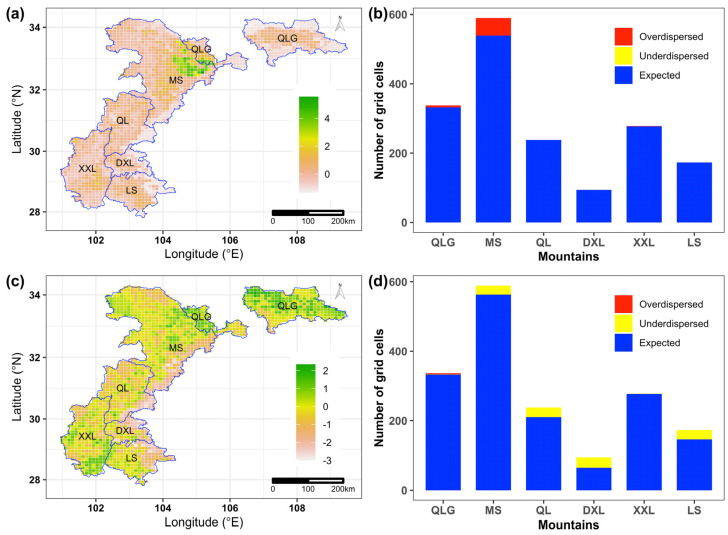
Spatial patterns of the standardized effect sizes (SES) for (**a**) functional diversity and (**b**) phylogenetic diversity in the whole study area and comparisons of (**c**) SES function diversity and (**d**) SES phylogenetic diversity among the six mountains in the study area: Qinling (QLG), Minshan (MS), Qionglai (QL), Daxiangling (DXL), Xiaoxiangling (XXL) and Liangshan (LS) mountains.

We found that no grid cells were functionally underdispersed in our study area, while about 3.39% (58) of grid cells in our study area were functionally overdispersed (Figure 3a). These grid cells were mainly distributed in the mountains of Minshan (51 grid cells), Qinling (6 grid cells) and Xiaoxiangling (1 grid cells) (Figure 3b). On the contrary, about 6.54% (112) of grid cells were found to be phylogenetically underdispersed, while only 0.18% (3) of grid cells in our study area were found to be phylogenetically overdispersed (Figure 3c). The grid cells that were found to be phylogenetically overdispersed were mainly distributed in Daxiangling (29 grid cells), Qionglai (28 grid cells), Liangshan (27 grid cells) and Minshan (26 grid cells) mountains, whereas all the grid cells that were found to be phylogenetically overdispersed were only distributed in Qinling mountains (Figure 3d).

### 3.3. Drivers of Taxonomic, Phylogenetic and Functional Diversity

**Figure 4:**
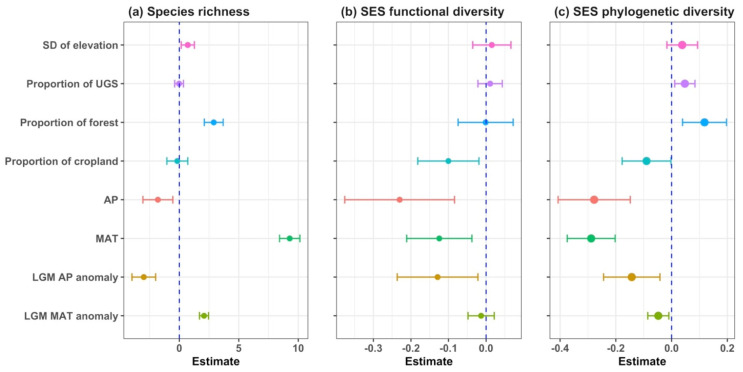
Coefficients of the generalized least squares regression models (Table 1) and their 95% confidence intervals for (**a**) species richness, (**b**) SES functional diversity and (**c**) SES phylogenetic diversity, respectively. SD: Standard deviation; UGS: urban green spaces; AP: annual precipitation; MAT: mean annual temperature; LGM: the Last Glacial Maximum.

Mammal species richness was mainly explained by SD of elevation, proportion of forest and all four climate variables (Figure 4a; Table 1). Specifically, mean annual temperature emerged as the strongest predictor, with a positive relation to species richness. LGM MAT anomaly, proportion of forest and SD of elevation also showed a positive relationship with species richness. On the contrary, LGM AP anomaly and annual precipitation showed a negative relationship with species richness. Mammal functional diversity was explained by the proportion of cropland, annual precipitation, mean annual temperature and LGM AP anomaly (Figure 4b; Table 1). All these predictors showed a negative relationship with SES functional diversity. Similarly, the above four predictors also showed a negative relationship with SES phylogenetic diversity in addition to LGM AP anomaly, while the proportion of UGS and forest showed a positive relationship with SES phylogenetic diversity (Figure 4c; Table 1).

## 4. Discussion

Understanding the patterns and drivers of different facets of biodiversity is crucial for biodiversity conservation under global environmental change [14,16]. In this study, we assessed the patterns and determinants of taxonomic, functional and phylogenetic diversity of mammals in the giant panda range. We found that there are mismatches of patterns and driving factors between functional diversity and the other two diversity facets. In particular, contemporary climate and land use conditions and climate anomalies between current and historical periods are the main drivers of mammal functional and phylogenetic diversity in the giant panda range. Moreover, mammal assemblages were functionally overdispersed and phylogenetically underdispersed in very few regions with high species richness. These findings can provide new insights into patterns, drivers and conservations for mammals in the giant panda range.

It is evident that large-scale variation in functional and phylogenetic diversity is related to variation in species richness to some extent [43,44]. Similarly, we found that mammals in Qingling, Liangshan and Daxiangling mountains harbored not only higher species richness but also higher functional and phylogenetic diversity, while Xiaoxiangling mountains harbored the lowest species richness and functional and phylogenetic diversity, highlighting the necessity of priority protection for Qingling, Liangshan and Daxiangling mountains. In particular, as a hotspot region with high priority for conservation, the Liangshan mountains should be included in the Giant Panda National Park, which aims to protect giant pandas and other species more effectively than protected areas [45]. Additionally, we also found that there are significant spatial correlations between species richness and the other two facets of mammal diversity, which are expected based on previous studies [5,44]. However, functional diversity was lower in most areas with high species richness and phylogenetic diversity in the giant panda range. Such cases of low functional diversity in species-rich areas are rare (e.g., [46,47]), as high functional diversity in species-poor areas is widely observed [10]. This rare pattern indicates that only those species with certain traits can persist in those regions, and these species are often evolutionarily far apart, suggesting that mammal assemblages in these regions were mainly structured by habitat filtering and competition [48,49].

Furthermore, our mapping of the spatial patterns of functional and phylogenetic diversity after controlling the effect of species richness also informs the potential mechanisms underlying observed mammal diversity. For example, we found overdispersed functional diversity but very limited variability in phylogenetic diversity in small areas of Qingling and Minshan mountains, suggesting that many species that persist in those regions have great variation in traits, but their relatives are close locally. These findings are consistent with previous studies [12,50], which showed that niche partitioning plays a key role in determining diversity patterns. For giant panda with its sympatric mammals, niche partitioning mainly occurs in morphology and other traits. Therefore, these regions allowed for the high richness of mammal species within a high diversification trait space. Actually, species packing in species-rich regions may also lead to assemblages with underdispersed phylogenetic diversity by promoting adaptive radiation [51].

Both climate and land use factors are important factors in determining the patterns of different facets of biodiversity [12,16,44]. Consistent with previous studies, our results show that land use, contemporary climate and climate anomaly between current and historical periods are all closely related to mammal taxonomic, functional and phylogenetic diversity in the giant panda range. Regarding land use factors, researchers have shown that the proportion of cropland and forest has an important effect on the distribution and habitat selection of mammals in the giant panda range, as these changes can have either a positive or negative effect on the habitat of mammals [32,52,53]. For example, Wei et al. [54] showed that increasing forest cover is a key natural resource for the recovery of giant pandas. Regarding temperature-related factors, however, both mean annual temperature and LGM temperature anomaly play completely opposite roles in affecting mammal diversity. Specifically, species richness was greater in the regions with higher mean annual temperature and LGM temperature anomaly, but functional and phylogenetic diversity were greater in the regions with lower mean annual temperature and LGM temperature anomaly. These findings suggest that only those species with wider climate niches and higher thermal tolerance can persist in those regions, and these species are often evolutionarily closely related, suggesting that contemporary temperature and long-term temperature stability play a crucial role in determining functional and phylogenetic diversity of mammals in giant panda range. Due to relatively low physiological tolerance to warming and narrow climate niche, many species in our study area are vulnerable to climate change [32], and consequently, future climate change would result in a decrease in the functional and phylogenetic diversity of mammals. Therefore, adopting effective conservation strategies to mitigate the negative effects of climate change and anthropogenic disturbances is essential to protect biodiversity and ecosystem functions in the giant panda range.

## 5. Conclusions

Overall, our study is the first large-scale assessment of patterns and determinants of different facets of mammal diversity in the giant panda range. Notably, species richness can closely mirror phylogenetic diversity but not the functional diversity of mammals in the giant panda range, although they show very similar patterns with respect to mountains and have a significant spatial correlation. Additionally, both land use factors and precipitation-related factors have a consistent effect on mammal diversity, while the effect of temperature-related factors varied among different facets of mammal diversity. More importantly, our results indicate that future climate warming and agriculture expansion may result in a loss of functional and phylogenetic diversity. Therefore, considering multiple facets of mammal diversity is essential to infer the underlying drivers determining biodiversity patterns and the effective conservation of these mammals. In particular, incorporating functional diversity into biodiversity studies is beneficial for conservation efforts, as the priority conservation regions identified by high species richness might not necessarily harbor high mammal functional diversity [55].

## Figures and Tables

**Table 1:** The output of the generalized least squares regression models for species richness, SES functional diversity and SES phylogenetic diversity, respectively. SD: standard deviation; UGS: urban green spaces; AP: annual precipitation; MAT: mean annual temperature; LGM: the Last Glacial Maximum.

Diversity Indices	Explanatory Variables	$\hat{\beta }$	SE	*t*	Pr (>|*t*|)
Species richness	Intercept	51.071	0.559	91.406	1.00 × 10^−90^
	SD of elevation	0.712	0.282	2.524	0.012
	Proportion of UGS	−0.019	0.187	−0.099	0.921
	Proportion of forest	2.895	0.404	7.162	1.18 × 10^−12^
	Proportion of cropland	−0.167	0.449	−0.373	0.709
	AP	−1.799	0.642	−2.804	0.005
	MAT	9.282	0.436	21.276	2.89 × 10^−89^
	LGM AP anomaly	−2.984	0.508	−5.875	5.07 × 10^−9^
	LGM MAT anomaly	2.080	0.194	10.695	7.01 × 10^−26^
SES functional diversity	Intercept	−0.124	0.086	−1.443	0.149
	SD of elevation	0.015	0.026	0.587	0.557
	Proportion of UGS	0.011	0.016	0.654	0.513
	Proportion of forest	−0.001	0.037	−0.032	0.975
	Proportion of cropland	−0.100	0.042	−2.414	0.016
	AP	−0.230	0.075	−3.087	0.002
	MAT	−0.125	0.044	−2.813	0.005
	LGM AP anomaly	−0.129	0.055	−2.354	0.019
	LGM MAT anomaly	−0.013	0.018	−0.742	0.458
SES phylogenetic diversity	Intercept	−0.263	0.06	−4.405	1.12 × 10^−5^
	SD of elevation	0.038	0.028	1.351	0.177
	Proportion of UGS	0.047	0.018	2.566	0.010
	Proportion of forest	0.118	0.04	2.933	0.003
	Proportion of cropland	−0.09	0.045	−2.014	0.044
	AP	−0.278	0.066	−4.205	2.75 × 10^−5^
	MAT	−0.288	0.044	−6.573	6.52 × 10^−5^
	LGM AP anomaly	−0.143	0.052	−2.772	0.006
	LGM MAT anomaly	−0.048	0.019	−2.489	0.013

## Data Availability

The original contributions presented in the study are included in the article, further inquiries can be directed to the corresponding authors.
